# Intracerebral hemorrhage in critically ill patients admitted in a general ICU of a hospital without neurosurgical service

**DOI:** 10.1186/2197-425X-3-S1-A982

**Published:** 2015-10-01

**Authors:** A Estella, T Rico Armenteros, M Jaen Franco, P Guijo, L Fernández Ruiz, M Recuerda Nuñez

**Affiliations:** Intensive Care Unit, Hospital del SAS de Jerez, Jerez de la Frontera, Spain

## Introduction

The type of ICU that admits patients with an intracerebral hemorrhage (ICH) varies considerably; some authors suggest that for critically ill patients with ICH admission in a neuro-ICU is associated with reduced mortality. Coordination with the reference centers is still necessary.

## Objectives

The aim of this study was to describe the clinical profile of patients with intracranial hemorrhage admitted to a general ICU and to analyze which patients are transferred to a hospital with neurosurgery department.

## Methods

Descriptive study in an ICU of a community hospital. Time of study was 20 months. Consecutive critically ill patients with ICH admitted in ICU were included. The variables analyzed were age, sex, medical history, APACHE II and Glasgow Coma Scores collected at the time of ICU admission, transfers to reference hospital and ICU length of stay.The data were analyzed using SPSS version 18 for Windows.

## Results

50 patients, 35 men and 15 women were included. Mean age was 64.12 ± 6.2 years. Most of patients had medical history of hypertension, 66.7%. A third of ICH patients were treated with antiplatelet agents and 16.7% were anticoagulated. 82% required mechanical ventilation and 54% were treated at admission with intravenous antihypertensive drugs. Maximum length of ICU stay was 27 days and 12% of patients required tracheotomy. 24 patients were accepted to transfer to a neuro-ICU at the reference hospital. We distinguish two subgroups according patients transferred to the reference hospital (24%), table 1 shows differences observed.

**Table 1:** Table 1

	Transfer to Neuro-ICU n:12	General ICU n:38
Age	52.6 ± 9.7	67.8 ± 11.2
Apache II at ICU admission	12.4 ± 6.4	23.5 ± 7.2
Glasgow coma score at ICU admission	7.5 ± 4.5	6.4 ± 3.7
Antiplatelet agents	8.3%	36.8%
Anticoagulation therapy	8.3%	18.4%
ICU lenght of stay (days)	2.2 ± 3.7	6 ± 6.39

*[Clinical characteristics ICH patients]*

## Conclusions

The predominant clinical profile was hypertensive male with requirements of mechanical ventilation and antihypertensive iv drugs at admission.

Age, GCS and Apache II at admission were significantly lower in patients accepted to transfer to the reference hospital for neurosurgical evaluation.

